# Editorial: Biophysics of co-translational protein folding

**DOI:** 10.3389/fmolb.2022.1110432

**Published:** 2022-12-07

**Authors:** Stephen D. Fried, Paula J. Booth

**Affiliations:** ^1^ Department of Chemistry, Johns Hopkins University, Baltimore, MD, United States; ^2^ T. C. Jenkins Department of Biophysics, Johns Hopkins University, Baltimore, MD, United States; ^3^ Department of Chemistry, King's College, London, United Kingdom

**Keywords:** protein folding, translation, ribosome, emerging methods, membrane translocation

Has the Protein Folding Problem been solved? This is an assertion that has now been made on at least two occasions: In 2005 once energy landscape theory had taken on the status as accepted theory to describe folding kinetics of model proteins (Wolynes, 2004), and more recently in 2021 with the advent of highly-accurate AI-based models for protein structure prediction (Jumper et al., 2021). Whilst there can be no doubt that structural prediction is a powerful tool that has transformed all facets of protein science, we would maintain that there are still a range of fascinating open questions surrounding how cells manage to produce extraordinary molecular architectures that are responsible for complex biological functions.

Formative protein folding studies concentrated on small, soluble, single-domain proteins that can reversibly refold from denaturant. These proteins were amenable to accepted physical theory (e.g., equilibrium thermodynamics), a handful of canonical spectroscopic methods (e.g., circular dichroism and bulk fluorescence), and could generally fit into a semi-universal framework (Maxwell et al., 2009). However, these models and methods have struggled to provide a framework for the folding of multi-domain proteins (see Rajasekaran and Kaiser) and of integral membrane proteins (see Mercier et al.), reviewed in two pieces in this Research Topic. What both classes of proteins have in common is a greater dependence to fold during their primary biosynthesis on the ribosome, known as co-translational folding–the focus of this Research Topic in *Frontiers in Molecular Bioscience*.

Co-translational folding represents a paradigm shift for protein folding research. Though the plausibility of its existence has been known as far back as 1963 (Zipser and Perrin, 1963), its importance for enabling biogenesis of larger and more topologically complex proteins has become appreciated only recently. It recasts the folding process as one quintessentially governed by kinetics, occurring on an evolving energy landscape that is continuously remodeled during chain elongation as well as by the idiosyncratic shape and environment of the ribosome itself.

Intimately probing this complex process involving myriads of tRNAs and translation factors has required the adoption and specialization of new tools. The commercial availability of *in vitro* translation has been one of the key developments that has democratized this line of research, and all three experimental contributions in this Topic availed of this technology. In addition to biochemical tools, research in cotranslational folding utilizes a distinct and relatively new suite of biophysical approaches, hence three of the contributions in this Topic focus on the application of these methods. Rajasekaran and Kaiser review the importance and usefulness of single molecule force spectroscopy measurements to probe partial domain-wise unfolding and refolding–particularly salient for complex proteins for which complete unfolding frequently cannot be reversed. Niwa et al. present a fluorescence correlation spectroscopy (FCS) approach to measure the diffusion coefficient of the co-translational chaperone, trigger factor (TF), both *in vitro* and *in vivo*. They show that TF maintains strong association with ribosome-nascent chains (RNCs) even in the presence of other chaperones, such as DnaK (Hsp70). Ataka et al. demonstrate the application of surface-enhanced infrared absorbance spectroscopy (SEIRAS) to probe secondary structure formation of membrane proteins, a class of proteins whose biogenesis is inextricably tied to translation, because their full-length forms are too hydrophobic to maintain in the cytosol. They report one bacteriorhodopsin that can form a correctly-folded tertiary structure in the membrane without the translocon, whereas three other proteins in the same family can form alpha helices but cannot insert into the membrane. The successful channel had the highest hydropathy index, suggesting that alpha helices with lower hydropathy might be more reliant on the translocon to mediate insertion. On a similar note, Mercier et al. provide a timely review on emerging details about the mechanism of polytopic membrane protein insertion in bacteria. With 166 references and authored by some leading figures, this piece can serve as an authoritative introduction to any newcomer to the membrane protein folding field. Significantly, this work reviews and highlights the mechanism and obligate clients of YidC, a more-elusive chaperone that operates with the core SecYEG translocon to mediate membrane protein biogenesis.

The first accepted piece in this Topic, authored by León-González et al., presents a detailed force profile analysis (FPA) for the repetitive ankyrin domain of the Notch receptor. Developed by von Heijne and coworkers (Cymer et al., 2015), FPA analyzes the capacity of various lengths of nascent chains to exert sufficient force to overcome an arrest peptide, and constitutes one of the signature methods of the co-translational folding field. In applying FPA to a repeat protein, the authors demonstrate clear evidence that peaks (lengths of nascent chain that apply substantial forces against the peptidyltransferase center) correspond to thermodynamically stable folding intermediates that were previously characterized in solution by equilibrium thermodynamics (Mello and Barrick, 2004). Hence, the authors show convincingly that interfaces form between repeats co-translationally.

Enough from us! Take a look at these excellent papers and make your own assessment. We hope that at the end you will share our enthusiasm for research into the biophysics of co-translational protein folding.
